# A case-control study to assess risk factors related to cholera outbreak in Addis Ababa, Ethiopia, July 2016

**DOI:** 10.11604/pamj.2019.34.128.17997

**Published:** 2019-11-05

**Authors:** Abduilhafiz Assen Endris, Musse Tadesse, Emana Alemu, Emmanuel Onuche Musa, Aschalew Abayneh, Zewdu Assefa

**Affiliations:** 1 Ethiopian Public Health Institute, Addis Ababa, Ethiopia; 2 World Health Organization, Regional Office for Africa, Brazzaville, Congo; 3 Ministry of Health, Addis Ababa, Ethiopia

**Keywords:** Cholera outbreak, risk factors, Addis Ababa

## Abstract

**Introduction:**

Suspected cholera cases were reported to the city administration health bureau in Addis-Ababa, Ethiopia on June 5, 2016 and it was laboratory confirmed by culture on 7 June 2016. The outbreak was declared by the government on June 8, 2016. The aim of this study was to rapidly investigate the outbreak epidemiologically and guide response activities in the affected Sub cities of Addis Ababa city administration.

**Methods:**

An unmatched 1:2 case-control study was conducted in six selected sub-cities of the city administration. Different laboratory tests were also done from suspected possible risk factors identified to support the epidemiological findings. A case was a patient greater than 5 years old, who developed acute watery diarrhea with or without Vomiting. Control was an individual greater than 5 years' old who stayed in the same township and did not suffer from cholera. A structured questionnaire was used to select cases and controls. Epi Info^TM^ statistical software was used to analyze the data. Results were presented in narratives, figures and tables.

**Results:**

The present study found that, the study participants who used street-vended water (Odds Ratio (OR)=10.4; 95% CI: 1.20-90.9), those who reported holy water sources use (OR=2.7, 95% CI: 1.45-5.04), eating raw meat (OR=7.75; 95% CI: 3.86-15.54) or roasted meat (OR=2.16; 95% CI: 1.19-3.93) and vegetable salad (OR=2.07; 95% CI: 1.14-3.76) were associated with contracting cholera infection. The likelihood of contracting cholera was significantly higher among those who ate food from street vendor sources (OR=5.32; 95% CI: 1.82-15.56) and those who practiced open defecation (OR=8.12; 95% CI (2.20-29.81). Having a latrine (OR=0.29; 95% CI: 0.12-0.69) and proper hand hygiene practice (OR=0.22; 95% CI: 0.14-0.38) were found to be protective against cholera infection.

**Conclusion:**

Improving awareness of the community by intensifying social mobilization activities through community participation in proper hygienic practice, proper waste disposal and latrine facility construction and utilization. Provision of safe water for the community by strictly conducting end-point assessment of water points and conducting a KAP assessment among food handlers to explore their knowledge and practices regarding safe food/drink handling and water treatment as well as initiate appropriate PH actions based on the findings which will be necessary for prevention of similar cholera outbreaks in the future.

## Introduction

Acute watery diarrhea is the passage of three or more loose or watery profuse diarrhea per day, in a period of 2 hours to 5 days. Other types of diarrhea include dysentery; and persistent diarrhea which lasts 14 days or longer. Patients presenting with diarrhea can also present with other symptoms such as vomiting, fever, and body weakness. Diarrhea causes loss of body fluid and electrolytes, which can result in dehydration. If dehydration is not corrected, death can be the result [1]. The causes of diarrhea can be bacterial, viral or parasitic. Bacteria causes of diarrhea include *vibrio cholera, escherichia coli, campylobacter jejuni, salmonellae and shigella species.* Viral causes of diarrhea include rotavirus, adenovirus, and coronaviruses. Parasitic causes of diarrhea include *giardia, entamoeba, cryptosporidium and the helminths* (Strongyloides, Schistosoma) [2]. Among these causative agents, acute watery diarrhea caused by vibrio cholerae serogroup O1 and O139 is the highly pathological [3, 4]. It is usually transmitted through faecally contaminated water or food and remains an ever-present risk in many countries [5]. Vibrio cholerae produces a toxin that stimulates the secretion of water and electrolytes in the intestinal tract. Patients with cholera may suffer from acute watery diarrhea, vomiting, and dehydration but rarely present with fever [6]. In Ethiopia it was indicated that, there was a cholera epidemic in 1990 which persisted with recrudescence of cases till 1998 [7]. Moreover, from July 2008 to June 2009 in Ethiopia, there were a total of 9485 cases and 193 deaths (with case-fatality rate 2.0%) of acute watery diarrhea in six regions including Addis Ababa [8]. Addis Ababa city is one of the city administrations of Ethiopia, according to the Central Statistical Authority report, the total population of the city administration is 3,384,569 [9]. During 2001, a total of 4000 cases and 40 deaths of cholera with a total case fatality rate of 0.33% were reported from the city administration.

### Statement of the problem and rationale

The first index case was detected and reported from private clinic (Geta Higher clinic) from Kolfe Keranyo sub-city Woreda 6 on June 5, 2016 by the managing physician through phone call. The second case was also reported from similar health facility on the same day. After the first suspected cholera case was reported from Kolfie Keranyo sub-city of Addis Ababa city administration, the cases were laboratory investigated for vibrio cholera and confirmed by culture on June 7 2016. The outbreak was officially declared by the government on June 8 2016. MOH led the response with support from partners through case management, surveillance and WASH interventions at all levels. On June 8 2016 the Addis Ababa city administration health bureau requested the Federal Ministry of Health, Ethiopian Public Health Institute and Public Health Emergency Management Center (FMOH-EPHI/PHEM) for investigation and response of the outbreak. A team from FMOH-EPHI/PHEM, Addis Ababa city administration health bureau, WHO and other partners which include health professionals and field epidemiology residents were deployed in the affected areas for investigation and strengthening prevention and control activities. Rapid assessment was conducted by the team deployed to the city administration with the aim of understanding the extent of the outbreak and identifies possible risk factors for the outbreak to support the preparedness towards implementing prevention and control activities. The assessment was done by visiting treatment sites and interviewing cases in CTCs and interviewing health professionals working at the CTCs and respective health system structures by using questionnaire and checklist prepared by the team. Possible risk factors implicated from initial assessments include drinking water from unprotected sources; rivers, springs and holy water sites; Open defecation due to lack of latrines, poor solid waste collection and disposal, Poor food hygiene, and overcrowding. Different factors including, the onset of the rainy season could lead to further spread of the outbreak. Besides this, large-scale population movements in and out of Addis Ababa, the capital city of Ethiopia increases the potential of further spread to other parts of the country and outside the country. Rapid identification of risk factors which were contributing for the outbreak was with paramount important to support the prevention and control activities for the outbreak.

**General objective:** to determine the factor(s) associated with increased risk of infection with cholera and to direct/guide/refine ongoing cholera outbreak prevention and control activities with a view to stopping transmission.

**Specific objectives:** to determine the possible cause(s)/risk factor(s) for the outbreak, to strengthening and/or guide prevention and control activities based on the finding and to make recommendations & report findings to decision makers

## Methods

**Study area and period:** the case-control study was conducted from 15^th^ July to 28^th^ July 2016 in six selected sub-cities of the city administration of Addis Ababa, the capital city of Ethiopia. Based on the 2007 census conducted by the Ethiopian national statistics authorities, the population of Addis Ababa is 3,384,569 million; all of the populations are urban inhabitants. The city has a total of 662,728 households with an average of 5.3 persons per a household. Most of the population of the city were followers of Ethiopian Orthodox religion (74.7%) followed by (16.2%) Muslim, (7.77%) Protestant and 0.48% Catholic [9]. According to the 2007 national census, 98.64% of the housing units of Addis Ababa had access to safe drinking water. From the total population 14.9% had access to flushing toilets, 70.7% pit toilets (both ventilated and unventilated), and 14.3% had no access to toilet facilities [9].

**Study design:** un-matched case-control study design was conducted to assess the risk factors of cholera infection. Different laboratory tests were also done from suspected possible risk factors identified to support the epidemiological findings.

**Study population:** the study population includes cases who meet the WHO standard case definition of the confirmed case as adopted by the Ethiopia Ministry of Health. Controls are defined as persons living in the same sub-city as a case but who had not suffered cholera until the date of the interview. New cases were identified from all CTCs and controls selected from all sub-cities in Addis Ababa with the continuing transmission.

Suspected case: a case of cholera should be suspected in: [10] an area where cholera is not known to be present, a patient aged 5years or more develops severe dehydration or dies from acute watery diarrhea; in an area where there is a cholera epidemic, a patient aged 5 years or more develops acute watery diarrhea, with or without vomiting. At the health post and at community levels, a suspected cholera case can be defined as follows: [10] any person 5 years of age or more with profuse acute watery diarrhea and vomiting

**Confirmed case:** a suspected case in which vibrio cholera O1 or O139 has been isolated from their stool [10].

**Sample size determination (assumptions):** least extreme odds ratio to be detected =2, hypothetical proportion of exposed controls =40%, hypothetical proportion of exposed cases =60%, confidence level =95%, Power =80%, number of cases =150, number of controls =150, total sample size =300 (Kelsey *et al.*) [11].

**Selection of cases:** the primary study subjects were newly confirmed cases of cholera admitted to the Cholera Treatment Centre (CTC) in the selected 6 hot spot sub-cities during data collection time. Line list in CTCs were used to identify newly admitted cases and systematically select cases for interview. Systematic selections of cases (every two cases from the line list) were used to ensure the random allocation of cases during the selection process. This principle was applied until the required numbers of cases were obtained. Cases were selected from the CTCs in six hotspot sub-cities during the study period were Kirkos CTC (Kirkos sub-city), Teklehymanot CTC (Lideta Sub-city), Woreda 10 CTC (Addis Ketam Sub-city), Alem Bank CTC (Kolfie Keranyo CTC) and Serti CTC (Akaki Kality Sub-city). A number of cases and controls collected were proportionally allocated for each CTCs based on case load they managed. Except kirkos and Serti CTCs, around 17 cases each were selected from each CTCs. A total of 16 cases were selected from kirkos and Serti CTCs each.

**Figure 1:**
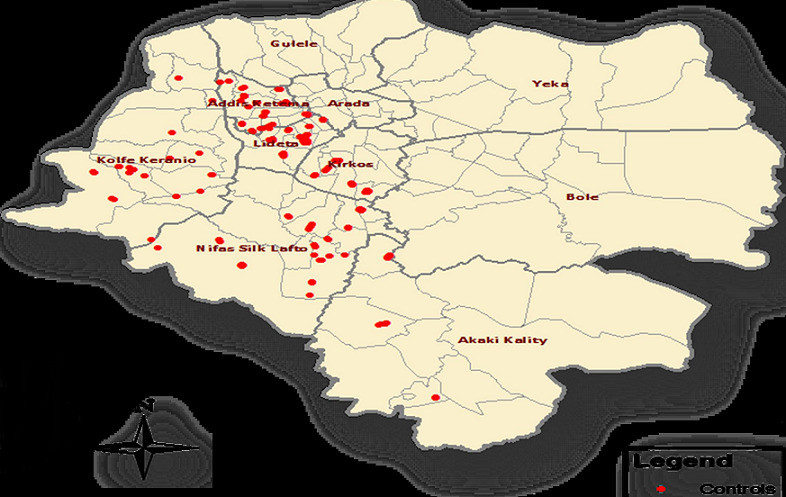
Distribution of selected controls at each sub-cities, August 4^th^ 2016

**Selection of controls:** to understand how rates of exposures to potential sources of infection differ between cases and uninfected persons, it was necessary to select controls from the same sub-city of the case. Controls were randomly selected as described below and asked for their consent to participate in the study. To maximize the power to show differences in exposures, two controls were recruited for each case. Prospective controls that have not had cholera illness were selected without conducting laboratory confirmation of the absence of cholera infection before selection. Controls were selected randomly. In the area where the case lives, two controls selected directly through a random selection process on site. For example, on arrival at the center of the selected village of the sub-city, go to the front of the nearest market or other reference and simply spin an empty bottle of coca-cola, and walk to the 3rd house on the street in the direction indicated by the bottle. At the selected household, all eligible household members needed to be listed and one randomly selected for the interview by using the lottery method. Only one member of a given household for control was selected. This was done repeatedly at the different places until the number of controls required was obtained. From the selected sub-cities 34 controls were recruited to the study except for kirkos and serti CTCs, 32 controls were allocated for them. The following figure shows the selected controls distribution (Figure 1).

**Exclusion criteria:** to maintain specificity, children under-5 was not included in this study. Persons who had recently recovered from cholera were also excluded.

**Data collection:** following the selection of cases and controls, and receipt of informed consent, cases (or their caregiver where the case is too ill to speak) and control subjects were interviewed using the questionnaire in appendix A and data were collected on some identifying information, demographic information, and date of onset and exposure to risk factors for infection including water and food sources, within three days prior to onset of disease. Two field epidemiology residents for each sub-city totally 12 residents were recruited as a data collector and 1 WHO surveillance officer was allocated for each sub-city totally 6 officers as supervisors for all hot spot sub-cities. Both the data collectors and supervisors were given 1-day orientation on their duties and responsibilities during the data collection process.

**Laboratory methods:** identifying the responsible sub-type pathogen and antimicrobial susceptibility test were done from cases included in this study and possible risk factors identified during environmental investigation. During this process, many human and environmental samples were taken and tests were done to support the epidemiological study and guide prevention and control activities. Stool samples were collected from the newly admitted cases and transported by using Cary Blaire transporting media to city administration regional laboratory for culture test. Thiosulfate-Citrate Bile-Sucrose (TCBS) agar and polyvalent antisera were used to culture vibrio organism and determine the serotypes respectively. During the antimicrobial susceptibility monitoring test, every 10^th^ culture positive samples was subjected for drug sensitivity test, which means 15 antimicrobial sensitivity tests were done for around 15 samples. Environmental samples were also collected and tested from possible risk factors identified.

**Prevention of cholera transmission among investigation staff:** all investigation staff were trained on infection control procedures including proper hand hygiene prior to implementation, in order to minimize their own risk of infection when in close contact with patients and during community visits.

**Data processing and analysis:** using un-matched case-control design, the study examined the differences in types of exposures between individuals with confirmed and epi-linked cholera and healthy controls in order to determine the risk associated with that exposure. A standard questionnaire was used to collect information. The purpose of this case-control study was to determine if a case was more likely to have been exposed to a risk factor in the three days before onset of illness than a similar uninfected person in the community. The study was done prospectively, enrolling new cases in the Cholera Treatment Centers (CTC) as they were identified. The controls who were persons not sick with cholera were chosen at random from the same community of residence of the cases. Data entry and analysis was conducted using the Epi-Info version 7.2. The associations between risk factors and infection among cases and controls were analyzed using chi-square statistics and expressed as odds ratios with 95% confidence intervals.

**Ethical consideration and Informed consent:** during the visit to both cases and controls, the purpose of the study was explained to all eligible subjects and their consent obtained by a trained member of the investigation team. Consent for children under the aged 5-15 years was obtained from their parents or guardians. Verbal assent was also obtained for children under 15 years.

**Dissemination of findings:** the findings of the study were interpreted and shared with the outbreak responders to assist in improving the outbreak response interventions.

**Further studies:** to complement this case-control study, additional epidemiological, microbiological investigations around new cholera cases, and environmental analysis including investigation of knowledge or supplies to practice sanitary food and drink preparation of food and drink handlers deserves further exploration. Furthermore, testing of areas around the infected households, communities and potentially contaminated water and food sources would be required.

## Results

### Epidemiological Findings

**Figure 2:**
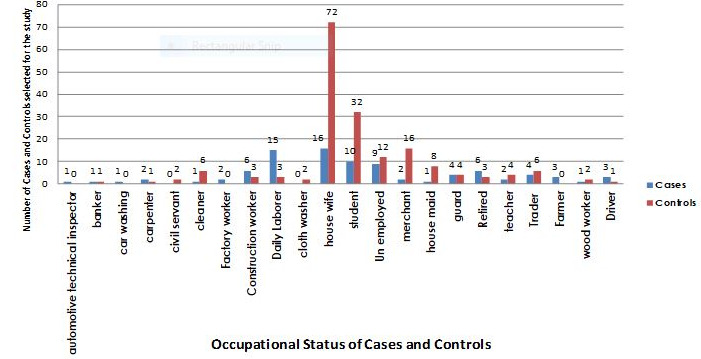
Distribution of cases and controls by occupational status, Addis Ababa, July 2016

**Demographic characteristics of cases and controls:** a total of 300 (100 cases and 200 controls) study participants participated in this study making a response rate of 100%. The age and occupation of the cases and controls were comparable but gender was not (corrected for during analysis). Most of the case respondents 60 (61.2%) and control respondents 143 (72.6%) were between 15-44 years age group. Almost 22% (22) of the cases and 20% (38) of the control's respondents were between 45 and 64 years of age. The median age for study participants of cases was 37 (range 11-80) and controls were 30 (range 10-90) (Table 1). The occupational status of the case respondents was also comparable with controls (Figure 2).

**Table 1:** Demographic characteristics of cases and controls, Addis Ababa, July 2016

Age Group (yrs.)	Cases	Controls
5-14	5 (5.1%)	3(1.5%)
15-44	60 (61.2%)	143 (72.6%)
45-64	22 (22.5%)	38 (19.3%)
>=65	11(11.2%)	13 (6.6%)
**Median Age (yrs)**	37 (Range 11-80)	30 (Range 10-90)
**Sex**		
Female	34 (34%)	157 (78.5%)
Male	66 (66%)	43 (21.5%)

**Clinical presentation of cases:** most of the cases wich were brought to the CTC center for treatment were brought with acute watery diarrhoea (99%), vomiting (80%), dehydration (67%) and others sign like fever and joint pain (4%) (Table 2).

**Table 2:** Clinical presentation of cholera cases, July 2016, Addis Ababa

Clinical Features	Number (%) of cases (N=100)
Acute watery Diarrhoea	100 (99%)
Vomiting	80 (80%)
Dehydration	67 (67%)
Others	4 (4%)

**Clinical presentation of cholera cases, July 2016, Addis Ababa**

**Drinking water exposure:** cases and controls were also compared with the drinking water exposure. Drinking street vended water and holy water were significantly associated with developing cholera. Those individuals who drank street vended water were 10.47 (1.20-90.90) times more likely to develop the infection than who did not with a p-value of 0.017. Similarly, those individuals who drank from holy water were 2.7 with CI of (1.45-5.04) more likely to develop the infection than those who did not drink with a p-value of 0.001 (Table 3). On the contrary treating water before drinking has a protective effect. Those who treated water before drinking had 0.17 (0.09-0.28) times protected than those who did not treat water before drinking with a p-value of 0.000 (Table 3).

**Table 3:** Distribution of cases and controls by drinking water sources, July 2016, Addis Ababa, Ethiopia

Exposure		Cases (n=100)	Controls (n=200)	Odds Ratio	95% CI	P-value
**Drinking Street Vended Water**	Yes	5 (5.0%)	1 (0.5%)	**10.47**	**1.20-90.90**	0.017*
	No	95 (95.0%)	199 (99.5%)			
**Drinking Holy Water**	Yes	26 (26.9%)	23 (11.5%)	**2.7**	**1.45-5.04**	0.001*
	No	74 (73.1%)	177 (88.5%)			
**Drinking Tanker Water (Roto)**	Yes	1 (1.0%)	1(0.5%)	2.01	0.12-32.48	1
	No	99 (99%)	199 (99.5%)			
**Drinking HH Tap-Water**	Yes	37 (37.0%)	82 (41.0%)	0.85	0.52-1.390	0.505
	No	63 (63%)	118 (59.0%)			
**Drinking Communal Tap-Water**	Yes	48 (48.0%)	109 (54.5%)	0.77	0.48-1.25	0.288
	No	52 (52%)	91 (55.5%)			
**Drinking Bottled-Water**	Yes	4 (4.0%)	17(8.5%)	0.45	0.15-1.37	0.229
	No	96 (96%)	183 (91.5%)			
**Drinking Borehole Water**	Yes	1 (1.0%)	0	-	-	-
	No	99 (99%)	200 (100%)			
**Drinking Spring Water**	Yes	5(5.0%)	0	-	-	-
	No	95 (95%)	200 (100%)			
**Treat Water Before Drinking**	Yes	27 (27%)	138 (69.0%)	**0.17**	**0.09-0.28**	0.000*
	No	73 (73%)	62 (31%)			

* P-value less than 0.05 is statistically significant

**Food and beverage exposures of cases and controls:** in regard to food and beverage exposures of cases and controls, eating raw meat, partially cooked meat and eating vegetable salad were significantly associated with developing the disease. Cases were more likely than controls to eat raw meat, partially cooked meat and vegetable salad. Those individuals who ate raw meat, partially cooked meat and vegetable salad had 7.75 (CI-3.86-15.54), 2.16 (CI-1.19-3.93), 2.07(CI-1.14-3.76) times more chance of developing cholera than their counterparts with a p-value of 0.000, 0.016 and 0.015 (Table 4).

**Table 4:** Distribution of cases and controls by food and beverage exposure, July 2016, Addis Ababa, Ethiopia

Exposure		Cases (n=100)	Controls (n=200)	OR	95% CI	P value
**Raw Meat**	Yes	35 (35.0%)	13 (6.5%)	7.75	3.86-15.54	0.000*
	No	65 (65.0%)	187 (93.5%)			
**Partially roasted meat**	Yes	26 (26.0%)	28 (14.0%)	2.16	1.19-3.93	0.016*
	No	74 (74 %)	172 (86%)			
**Vegetable salad**	Yes	26 (26.0%)	29 (14.5%)	2.07	1.14-3.76	0.015*
	No	74 (26.0%)	171 (85.5%)			
**Fruits**	Yes	28 (28.0%)	65 (32.5%)	0.81	0.48-1.57	0.427
	No	78 (78.0%)	135 (67.5%)			
**Fish**	Yes	0(0.0%)	2 (1.0%)	-	-	-
	No	100 (100%)	198 (99.0%)			
**Un boiled fresh milk**	Yes	9 (9.0%)	7(3.5%)	2.73	0.98-7.55	0.046*
	No	91 (91%)	193 (96.5%)			
**Shameta**	Yes	3 (3.0%)	2 (1.0%)	3.06	0.50-18.6	0.338
	No	97 (97.0%)	198 (99.0%)			
**Besso**	Yes	8(8.0%)	15(7.5%)	1.07	0.44-2.62	0.878
	No	92 (92.0%)	185 (92.5%)			

* P-value less than 0.05 is statistically significant

**Food outside of the home:** among the study participants who ate outside their homes, the individuals who ate from street vendors and restaurants were significantly associated with developing the infection. Cholera cases were more likely than controls to have eaten food sold by street vendors and restaurants. Individuals who ate street vended and restaurants food were 5.32 (1.82-15.56) and 2.7 (1.36-5.32) times more likely to develop cholera than those who did not eat with a p-value of 0.001 and 0.003 (Table 5).

**Table 5:** Distribution of cases and controls with exposure status outside of the Home, July 2016, Addis Ababa, Ethiopia

Exposure place		Cases (n=100)	Controls (n=200)	OR	95%-CI	P value
**Street Vendor**	Yes	12 (12.0%)	5 (2.5%)	5.32	1.82-15.56	0.001*
	No	88 (88.0%)	195 (97.5%)			
**Restaurant**	Yes	21 (21.0%)	18 (9.0%)	2.7	1.36-5.32	0.003*
	No	79 (79.0%)	182 (91%)			
**Hotel**	Yes	10 (10.0%)	11 (5.5%)	1.9	0.78-4.66	0.15
	No	90 (90.0%)	189 (94.5%)			
**School/Work cafeteria**	Yes	8 (8.0%)	9 (4.5%)	1.84	0.69-4.93	0.216
	No	92 (92.0%)	191 (95.5%)			
**At a gathering**	Yes	13(13.0%)	37 (18.5%)	0.66	0.33-1.30	0.228
	No	87 (87.0%)	63 (81.5%)			

* P-value less than 0.05 is statistically significant

**Hygiene and sanitation:** in regards to Hygiene and sanitation practices among cases and controls, cases were more likely to practice open defecation, having a latrine and hand hygiene were a protective effect. Those individuals who practice open defecation were 8.12 (CI-2.20-29.81) times more likely to develop cholera than those who did not practice open defecation. Those who used the latrine, washed hands before eating and defecation were 0.29 (CI-0.12-0.69), 0.32(CI-0.19-0.52) and 0.22 (CI-0.14-0.38) more protective effect than those who did not with a p-value of 0.004, 0.000 and 0.000 (Table 6).

**Table 6:** Distribution of cases and controls by different hygiene and sanitation practice, July 2016, Addis Ababa, Ethiopia

Hygiene/sanitation	Cases (n=100)	Controls (n=200)	OR	95% CI	P value
**Open defecation**	11 (11.0%)	3 (1.5%)	**8.12**	**2.20-29.81**	0.001*
**Defecation in river**	1 (1.0%)	1 (0.5%)	2.01	0.12-32.48	1.000
**Latrine**	86 (86.0%)	191 (95.5%)	0.29	0.12-0.69	**0.004**
**Wash Hands +Soap Before Eating**	43 (43.0%)	141 (70.5%)	0.32	0.19-0.52	**0.000**
**Wash Hands + Soap After Defecation**	44 (44.0%)	155 (77.5%)	0.22	0.14-0.38	**0.000**
**Dispose refuse in River**	3 (3.0%)	5(2.5%)	1.20	0.28-5.15	1.000
**Dispose refuse in Open pit**	12 (12.0%)	25 (12.5%)	0.95	0.46-1.99	0.901
**Open dumping**	9 (9.0%)	20(10.0%)	0.89	0.39-2.03	0.782
**Dispose refuse by burning**	7 (7.0%)	0	-	-	-

* P-value less than 0.05 is statistically significant

**Laboratory surveillance and findings:** vibrio cholera 01 (ogwa) were identified as the responsible pathogen for the outbreak from study samples included in this study. During the antimicrobial susceptibility monitoring test, every 10^th^ culture positive samples was subjected for drug sensitivity test, that means 10 antimicrobial sensitivity tests were done for around 10 samples. The result showed that, except ampicillin the bacteria was sensitive for cotrimoxazole, erythromycin, tetracycline and doxycycline. From 695 environmental tests done from different sources like food and drink establishments, holy water sites and other suspected potential sources during the study period, 5.9% of them were tested positive for RDT and culture test. Besides these, meats tested from 12 different food and drink establishments and 11 holy water samples from two holly water sites were positive for the causative agent of the outbreak. Regarding water chlorine residual tests done at different spots (source, pipe line and at house hold), the residual chlorine in several spot checks of public taps were found below the minimum requirement level expected to be during outbreak which is 0.5. There were different food and drink establishments including abattoirs inside the city administration from which cluster of cases were reported. To prevent and control the dissemination of the outbreak and prevent extra, exposure from the reported establishments extensive screening was done for the food handlers and the environment of the establishments. From 964 screened butchers of abattoir A, 33 of them were tested positive. From the 33 tested positive for RDT, 18 of them were confirmed by culture.

## Discussion

As of 4^th^ August, the cumulative number of cases reported was 5.879 (AR=0.17%) and 12 deaths (CFR 0.20 %). Among them, 64% were males and 36% were females. All the 10 sub-cities and all Woredas in the sub-cities had reported cases. Most of the cases affected by this outbreak were from the 15-45 age groups, greater than 60% of the cases admitted and treated were within this age group. 139 (2.4%) were less than 5 years of age, 307 (5.2%) were within 5-14 years and 1838 (31.3%) were greater than 45 years of age. Of the total cases, 17.4% occurred among daily laborers, housewives (14.6%) and unemployed (10%). Forty-three percent of the cases were reported from three sub-cities (Kolfe Keranyo, Nifas Silk and Addis Ketema). The Attack rate (%, per 100 population) for the most affected sub cities were Nifas silk Lafto (AR=0.32), Akaki Kality (AR=0.32) and Addis Ketema (AR=0.28). 1.529 (26%) of the reported cases were presented with severe dehydration.

This study revealed lower attack rate (0.17%) and case fatality (0.20%) which is very low compared with the previous outbreak in the city administration and other outbreaks in different countries like Zimbabwe, Haiti and Sierra Leone [8, 12-15]. This may be related to the relative improvement on public awareness and hygiene and sanitation of the town besides previous experience and improvement on clinical management of cases. The case and control groups were unmatched with respect to age, gender, household size and other characteristics. Among the possible source of infections identified, drinking water source, food and beverage exposure, food consumption outside home, hygiene and sanitation were considered in this study so as to identify the risk factors for contracting cholera outbreak. Besides this, the epi-curve of the outbreak revealed that, the outbreak follows propagated epidemic pattern which probably indicates that there was also person to person transmission of the disease from diseased patients through above listed risk factors. This type of epidemic pattern for this outbreak was recorded in many previously affected countries like Haiti and Zimbabwe [13, 15]. In this study, drinking street vended water and Holy water was a statistically significant risk factor of contracting cholera. This finding is comparable with the findings of cholera outbreak investigation in Sierra Leone and watery diarrhea in Zimbabwe by the year 2012 and 2011, respectively [12, 13]. Both cases and controls had very high access to improved water sources, with public taps representing the most common type of improved water source, but the residual chlorine in several spot checks of public taps were found below the minimum requirement level. This finding suggests that either chlorine introduced into the water system at its origin was not reaching some water points in the area sampled, or that chlorination may not have been occurring.

On the other hand, sewage has entered networks of water pipes through cross-contamination with sewer pipes in some areas. The lack of chlorine and unhygienic water storage at household level could make this and other improved water sources susceptible to contamination with disease-causing agent like vibrio cholerae. Overcrowding, competing priorities and limited resources have resulted in poor hygiene and sanitation conditions. These conditions combined with low chlorination rates create an environment highly vulnerable to cholera transmission. An evaluation of the integrity of water distribution networks and chlorination practices would be useful to identify and resolve potential deficiencies. In the mid-long term, improvement and expansion of the water and sanitation infrastructure will help to prevent future epidemics of cholera and other waterborne diseases. Regarding sanitation practices, this study revealed that those individuals who practice open defecation were 8.12 times more likely to develop cholera than those who did not practice open defecation. Besides this, using latrine regularly, washing hands before eating and defecation had shown more protective effect. This study finding on sanitary practice was compatible with other studies done in different countries. Several studies have shown the health benefits of hand washing with soap and water and using latrine has significant role in the prevention and control/spread of communicable diseases [16-18]. In this study, a wide range of food items were tested to see if they were associated with the risk of cholera infection. The collected data indicate that eating raw meat products (like raw meat and dullet) and vegetables was a risk factor for cholera infection. Even though this study was not designed to determine whether street vendors had the knowledge or supplies to practice sanitary food and drink preparation, this area deserves further exploration. Information, Education and Communication (IEC) campaign focused on the importance of safe food handling practices and the importance of consuming water that has been treated with a chlorine product are core public health prevention activities.

## Conclusion

The age and occupation of the cases and controls were comparable but the gender was not (corrected for during analysis). Statistically significant risk factors for cholera found in this study include: drinking street vended water and holy water, eating raw meat, partially roasted meat, vegetable salad or unboiled fresh milk; food consumption at a street vendor or restaurant, lack of access to latrine (open defecation), most common type of water source is tap water, but end-point water quality needs to be closely monitored (eg residual chlorine testing) and evidence that population is responding to household water treatment and hand washing advice. We will recommend that they: conduct repeated end-point water sampling and testing including holy water sites to ensure improved water quality, reinforce point-of-use drinking water treatment to ensure safe drinking water, conduct a KAP study among food handlers to explore their knowledge and practices regarding safe food/drink handling and water treatment, and initiate appropriate PH actions and intensify social mobilization campaigns on hand washing, water treatment and sanitation.

### What is known about this topic

- Causes for Acute Watery Diarrhea can be bacterial, viral or parasitic;
- Known risk factors for cholera were drinking water from contaminated unprotected sources; like rivers, springs and other water sites;
- Prevention strategies for cholera were regularly treating and regulation on water and food sources for the community.

### What this study adds

- Prevention strategies should address the public by increasing their knowledge on treatment water before use by intensified social mobilization activities;
- Programmatic activities should also address segment of populations which are at higher risk for the disease and food handlers;
- Regular monitoring at a point in use for drinking water and food and drink establishments should be given higher priority.

## Competing interests

The authors declare no competing interests.
